# Sirolimus: A Rescue Drug to Control Complications of Extensive Venous Malformation

**DOI:** 10.1055/s-0040-1716895

**Published:** 2020-12-15

**Authors:** Mohamed Aly Abdelbaky, Iman Ahmed Ragab, Amr AbdelHamid AbouZeid, Shaimaa Abdelsattar Mohammad, Mohamed Moussa Dahab, Mohammed Elsherbeny, Hatem Abdelkader Safaan

**Affiliations:** 1 Department of Pediatric Surgery, Ain Shams University Faculty of Medicine, Cairo, Egypt; 2 Department of Pediatrics, Ain Shams University, Faculty of Medicine, Cairo, Egypt; 3 Department of Radiodiagnosis, Ain Shams University Faculty of Medicine, Cairo, Egypt

**Keywords:** multidisciplinary team, vascular anomalies, vascular malformations, venous malformations

## Abstract

Venous malformations represent a major sector of vascular anomalies. Most cases are asymptomatic or subclinical; however, large extensive lesions can cause severe disability and sometimes mortality. In this report, we present a successful case of sirolimus treatment in managing an extensive venous malformation in the pelvis of a 21-month-old boy who presented with life-threatening complications. With a history dating since the day 2 of life, the patient suffered from chronic bleeding due to scrotal skin ulcerations, in addition to recurrent attacks of severe bleeding per rectum necessitating hospital admission and blood transfusion (three attacks since the age of 7 months). Pelvic magnetic resonance image showed the typical findings of extensive venous malformation involving the pelvis, perineum, scrotum, and extending to the gluteal region. The lesion was seen totally encasing the anorectum with marked thickening of their walls almost occluding their lumen.

Oral sirolimus (2 mg/m ^2^ ) was started with a target blood trough level of 5 to 10 ng/mL. Over a follow-up period of 5 months, there was obvious clinical improvement that included healing of skin lesions (scrotal ulcer) with complete re-epithelialization, absence of bleeding per rectum with improvement of constipation, and rise of hemoglobin level from 7.5 to 11.5 g/dL.

## Introduction

Venous malformations (VM) represent a major sector of vascular anomalies that can exist either in an isolated form or in combination with other vascular malformations (complex vascular malformations). 1 VM result from abnormal endothelial cell morphogenesis leading to formation of nonfunctioning dilated networks of venous lakes with dysplastic communication to the normal venous system. 2 Most cases are asymptomatic or subclinical; however, large extensive lesions can cause severe disability and sometimes mortality. 1

International acceptance of a modern classification and the development of interdisciplinary teams to manage vascular anomalies represent great achievements in the field. 3 The recent advances in studying the molecular genetics of vascular anomalies have paved the way to enter a new era with expanded therapeutic options. 4 Sirolimus, also known as rapamycin, is a serine/threonine kinase which regulates the signaling pathway PI3K/AKT/mTOR controlling angiogenesis and cell growth. 5 Sirolimus was approved as an immunosuppressant in 1999 and was used in renal transplantation. 5 Owing to its antiangiogenic and antiproliferative properties, sirolimus has been successfully used in small case series to improve symptoms of severe complicated vascular anomalies. 6 7

In this report, we present a successful case of sirolimus treatment in managing an extensive venous malformation in the pelvis of a 21-month-old child who presented with life-threatening complications.

## Case Presentation

**Fig. 1:**
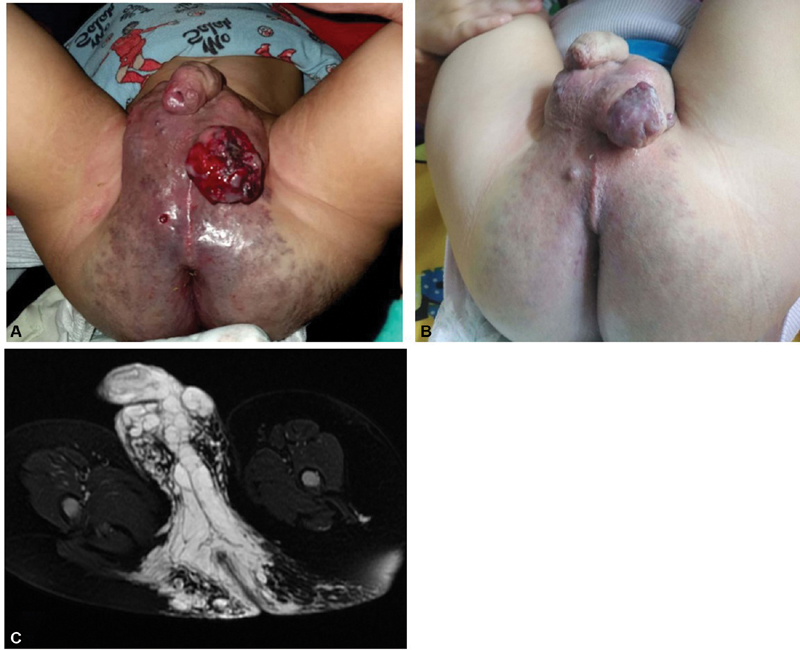
A 21-month-old boy presenting with extensive venous malformation in the perineum and pelvis. ( **A** ) Complicated presentation with chronic bleeding from a fungating skin ulcer. ( **B** ) After 3-month treatment with oral sirolimus showing marked improvement of skin lesions. ( **C** ) Pelvic magnetic resonance imaging (T2WI with fat suppression): axial cuts through the perineum to demonstrate the extension of the lesion that appeared as hyperintense dilated venous lakes.

A 21-month-old boy was referred to our vascular anomaly clinic with extensive vascular malformation in the perineum and pelvis ( Fig. 1 ). At day 2 of life, he had bleeding from a scrotal lesion for which a trial of surgical ligation failed resulting in a fungating skin ulcer with persistent chronic bleeding ( Fig. 1A ). He had a history of recurrent attacks of severe bleeding per rectum necessitating hospital admission and blood transfusion (three attacks since the age of 7 months). Attacks of bleeding per rectum accompanied severe straining at defecation. Before referral to our clinic, the patient was given a generic diagnosis of hemangioma and started oral propranolol with no improvement.

**Fig. 2:**
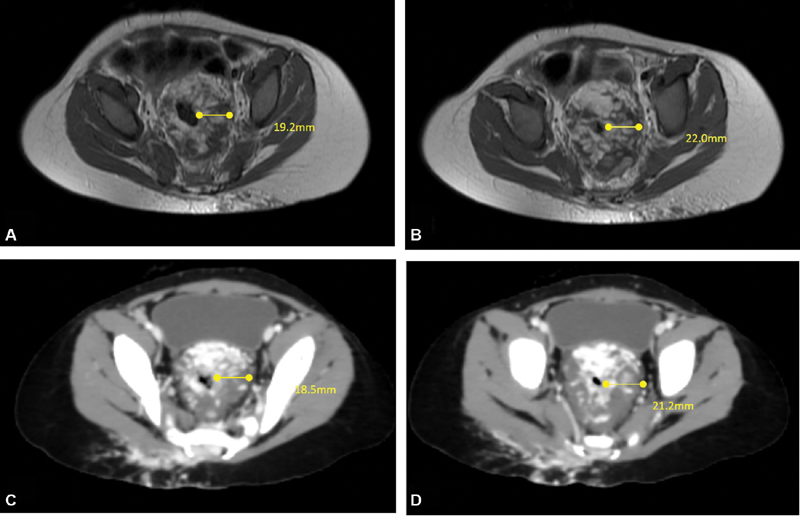
Axial imaging demonstrating the deep pelvic component of the venous malformation. The venous malformation is seen totally encasing the anorectum with marked thickening of their walls almost occluding their lumen. The upper row ( **A** , **B** ): magnetic resonance imaging study performed before referral to our vascular clinic. The lower row ( **C** , **D** ): computed tomographic angiography performed 2 months later. Note the patchy and delayed contrast enhancement of the lesion typical for venous malformation. By comparing both studies (upper row with lower row), no appreciable changes could be detected in the deep pelvic component of the lesion in contrary to the external clinical improvement.

Upon referral, his laboratory investigations showed moderate-to-severe anemia with elevated D-dimer levels (hemoglobin: 7.5 g/dL, mean corpuscular volume: 62.5fL, platelets: 197 × 10 × 9/L, prothrombin time: 12.7 seconds, activated partial thromboplastin time: 39.4 second, fibrinogen: 2.8 g/L, D-dimer 3,490 µg/mL fibrinogen equivalent unit). He had a pelvic magnetic resonance imaging (MRI) study already performed before referral that showed the presence of extensive venous malformation involving the pelvis, perineum, scrotum, and extending to the gluteal region ( Fig. 1 ). The venous malformation consisted of cystic spaces exhibiting hyperintense signal on T2WI, intermediate signal on T1WI, with typical delayed patchy contrast enhancement ( Fig. 2 ). The lesion was seen totally encasing the anorectum with marked thickening of the rectal wall almost occluding the bowel lumen ( Fig. 2 ). The patient was admitted on intravenous tranexamic acid and intravenous iron replacement (once). Laxatives were prescribed to help evacuation of stools, while the possibility of colostomy was discussed with parents who preferred to avoid this option. As the lesion was so extensive and difficult to treat by conventional therapeutic modalities, besides the presence of life-threatening complications (severe bleeding per rectum necessitating blood transfusion), we decided to start oral sirolimus. The dose was intended to be given on 0.8 to 1 mg/m ^2^ every 12 hours, yet due to unavailability of the solution form of the drug, it was changed to 2 mg/m ^2^ (once/day); this dosing approach is used in immune cytopenia. 8 The target blood sirolimus trough level was 5 to 10 ng/mL waiting for clinical/radiological response. In case of poor response, we would have incremented the dose to target a higher trough level (10–15 ng/mL). 9

After 1 week of hospital admission and starting oral sirolimus, the patient showed clinical improvement. Bleeding decreased from the scrotal ulcer, and there was improvement of straining at defecation with absence of rectal bleeding. The patient was discharged with hemoglobin 8.7 gm/dL to continue oral sirolimus at home in addition to oral iron replacement and laxatives. Over a follow-up period of 5 months, the skin lesions showed continuous improvement ( Fig. 2B ) with no detectable side effects for the drug. Progressive improvement was obvious for the medical staff at the clinic and well appreciated by parents as well. Clinical improvement included healing of fungating skin lesion (scrotal ulcer) with complete re-epithelialization, absence of bleeding/rectum with marked improvement of constipation (laxatives were occasionally required), rise of hemoglobin level reaching 11.5 g/dL, and lastly, the child could walk for the first time. It seems that the extensive lesion in the pelvis, perineum, and gluteal region was causing painful limitations for movement.

A follow-up imaging (computed tomographic angiography) was performed 2 months after the start of sirolimus treatment ( Fig. 2 ). This was ordered by our colleagues (interventional radiologists) to assess the possibility of injection/embolization of the lesion. Contrarily to the marked clinical improvement (regarding pain, mucosal bleeding, and skin changes), no appreciable changes could be detected in the deep pelvic component of the lesion ( Fig. 2 ).

## Discussion

VM are the most common peripheral vascular anomalies ranging from simple vessel dilatation to complex spongiform venous lakes infiltrating through multiple tissue planes. 10 Aching pain, disfigurement, and localized intravascular coagulopathy are more common complications with large complex extensive lesions. 1 11

Although spontaneous bleeding is uncommon with VM, 1 this case presented with repeated attacks of bleeding per rectum and chronic bleeding from skin lesions. Deficient vascular smooth muscle may be responsible for the gradual expansion of these abnormal venous lakes, 10 while their submucosal location made them subjected to repeated trauma at defecation. Associated coagulopathy (as indicated by elevated D-dimers) can increase the severity of bleeding in these cases. 1

There is a growing evidence on the effective role of mTOR inhibitors (sirolimus) in treating vascular anomalies. 4 Several reports have shown sirolimus to improve symptoms related to extensive vascular malformations that included pain, mucosal, and skin changes. 6 7 12 Moreover, its impact on hemostasis was recently reported where 12 patients with combined slow-flow vascular malformations and three pure VM had significant decrease in D-dimer levels following treatment with sirolimus. 9 Also, Freixo et al reported on the decrease in the size of lesions in 88.9% of treated cases. 13 However, sometimes reduction in the size of lesions may be inconsistent with symptomatic improvement. 14 Also, a percentage of cases would still have poor survival despite clinical improvement, while other types of malformations (arterio-VM) do not respond to sirolimus. 4 Treatment options for AVM remain limited and include angiographic embolization with or without complete surgical resection. 15

Some authors observed a better response with initiation of sirolimus at a younger age. 16 The duration of treatment is still indeterminate. Most of the publications have used a minimal duration of 6 to 12 months prior to evaluation, yet many patients had extended use of sirolimus beyond 30 months. 17 The effect of sirolimus on improving the quality of life in this case report was quite evident. For about 2 years, the patient suffered from chronic bleeding due to skin lesions and repeated attacks of massive mucosal bleeding per rectum. Clinical improvement was obvious within a week from start of sirolimus, and the patient could walk for the first time 4 months later.

Efficacy of the medication was evaluated based on physical (clinical appearance of skin lesions), functional (organ dysfunction, mobility restraint, pain, and bleeding), and biological (measurement of coagulation parameters). Best timing for radiological response is variable; in some publications, evaluation was done after 1 year of treatment. Among 16 patients evaluated radiologically after 12 months of treatment, T2 MRI-weighted sequences showed a size reduction in 43.7%, and despite clinical improvement, size progression was observed in 18.7%. 7 In a precedent study, stable disease was found in 63% at 6 months evaluation despite clinical response of 83%. 16 In several previous work, the clinical response outweighs the radiological response. 16 17 Some researchers attributed the radiological response to improvement in the lymphatic component and less inflammatory changes. In our proband, with a predominant venous malformation, a reduction in angiogenesis and congestion and less thrombophlebitis might have contributed to an earlier clinical response than radiological reduction in volume of malformation.

In such extensive VM, sirolimus can be used effectively to control complications as an adjunctive to other therapeutic options (multimodality treatment). The scrotal lesions can be partially excised surgically (debulking) with or without preoperative percutaneous “glue” embolization. 1 On the other hand, the deep extensive pelvic lesions encasing the anorectum may not be accessible for surgical excision. Therefore, repeated sessions of intralesional injection of sclerosing agents (e.g., bleomycin) would be a more practical option for the deep component of the lesion.

## Notes

New Insights and the Importance for the Pediatric Surgeon Sirolimus is an example of new treatment modalities recently introduced in the management of vascular anomalies that have favorably changed the course of disease.
